# Cases from Dr. Hamilton’s Clinic at the Buffalo Hospital of the Sisters of Charity

**Published:** 1859-04

**Authors:** J. Boardman


ART. V.—*Cases from Dr. Hamilton's Clinic at the Buffalo Hospital of the Sisters of Charity.* Reported by J. BOARDMAN, M. D.

*Fracture of the Femur.* D. L., aged 15 years, while playing upon a hand-car on the railroad, fell in front of the car and was struck by it on his left thigh. He was immediately brought to the hospital.

On examination three hours after the accident, discovered a fracture of the left femur, a little above the union of the lower third with the upper two-thirds of that bone. There was but little swelling of the limb, and the skin did not appear to be much bruised. The limb was shortened, distinct crepitus could be felt, and motion was perceptible at that point. The fracture could be reduced with comparative ease, but extension being removed, the ends of the bone would slip past each other.

His limb was dressed in the usual manner, with Desault's straight splint. Only slight swelling followed, and the boy being very quiet, the bandages did not become displaced, and therefore were left untouched until the seventeenth day, except they were tightened by being sewed over and over with a strong thread. At this time, Dr. Hamilton took the dressings from the thigh, examined the limb, and found that union had taken place, though as yet the bone was not firm. He measured the limb and re-dressed it before the class. The points taken for this measurement, were the anterior superior spinous process of the ilium, and the lower edge of the patella; also the lower ends of the external and internal malleoli of both legs. The left femur was found to be three-quarters of an inch shorter than the right.

In measuring a limb, the only reliable way is to take fixed and corresponding points upon both legs and carefully mark them with ink, or the tinct. of iodine, and when sure that the points are correctly marked, then with a string, or tape-line, compare the distances on one leg, with the distances on the other. It is best to select two or three different points upon each limb; and if the result is the same when the different measures are compared, it is a proof that the result is correct. It has been, and still is, the custom with a few physicians, to bring both heels together, the patient lying on his back in bed, and from the fact that both heels are brought together, they firmly believe that the result of their treatment is perfect. But this mode of measuring is exceedingly incorrect; for almost every one can easily, without any perceptible movement of the pelvis, cause either leg at will to appear longer than the other.

Dr. Hamilton has carefully measured a large number of fractured femurs

—cases treated not only by himself and by other American surgeons, but also by European surgeons—and union has never taken place without any shortening. The usual shortening in oblique fractures of the femur, is from one-half to three-quarters of an inch. In children, the femur often unites with no apparent shortening. The degree of lameness, however, is not in proportion to the amount of shortening. I have seen a person walk quite lame, whose leg was not shortened more than half of an inch, and again, I have seen a patient hardly appearing lame with one femur one and a-half inches shorter than the other. It is not an uncommon occurrence to find a person, even years after a fracture of this bone, with a shortening of one-half to three-quarters of an inch, entirely unaware of any difference in the length of his legs.

Dr. Hamilton, in re-dressing the limb, called the attention of the class especially to the various *little things*, as they might be named, but which add so much to the comfort of the patient, often prevent a great deal of anxiety on the part of the attending surgeon, and assist greatly in accomplishing a good result.

On being called to a case of supposed fracture of the femur, the surgeon should carefully remove, if it has not been done, all of the clothing from the limbs; examine and accurately determine the nature of the accident. If the case proves one of fracture of the femur, he should carefully ascertain the exact point at which the bone is broken, and also the character of the fracture, if possible; also the condition of the skin, for if the latter is much injured, his dressings must be so arranged as to avoid pressure over the wounded parts. In doing this he will be obliged to hurt the patient, but good surgeons will be careful in regard to giving pain to any one under his care. The day has gone past, when the worth of a surgeon is to be estimated by his rudeness, or his seeming indifference to suffering, and now he is deemed worthy of confidence whose hand is the lightest, and who carefully avoids giving his patient unnecessary pain.

After learning all that he can by his examinations, let the surgeon prepare the splints that he will wish to use, see that he has a number of pads made, not only for the splints, but also to protect certain parts of the limb. These pads should be made of cotton batting, or wadding, carefully arranged, smooth, free from all lumps, and sewed in a piece of cotton cloth, by lapping one edge over the other so that there may not be a hard seam. If loose cotton is used without being placed in cloth, it is liable to become displaced under the splint, and the surgeon cannot as readily ascertain whether the splint is entirely and evenly protected from undue pressure at any point. If loose cotton is employed, the dressings are apt to look slovenly, and also the limb will require much more frequent dressing. Dr. Hamilton often remarks, that the work of a *good surgeon* will be neat in appearance; also that a broken bone slovenly dressed, will, in most instances, give poor results. See that the bandages are prepared and properly rolled; also let the surgeon prepare four strips of adhesive plaster, two inches wide, and long enough to extend from the knee to at least ten inches beyond the foot. Let him prepare a perineal band. The one generally used by Dr. Hamilton for the last few years, is made as follows: a piece of strong cotton cloth, one and three-quarters of a yard in length, and four inches wide, is taken, and in the center is placed a few folds of wadding, or batting; over this is laid the half of a newspaper that has been folded so as to form a strip about one and a-half inches wide. The cloth is then folded and sewed in the same manner as in the pads, and when complete forms a band about two feet, the center of which is padded, while the ends are free. The surgeon cannot be too careful in seeing that this band is perfectly free of all lumps, or ridges, and that even the lapping of the cloth is on the outside, over the paper, for he is obliged to rely upon this for his counter-extension, and if the perineum becomes ulcerated, it will be the cause of trouble, to both him and his patient. Experience has proved that a newspaper folded in this manner, is of great assistance in preventing the wrinkling or cording of this band; and it also possesses this great advantage over many other substances, that it may be found in almost every place in this country, at least, there is hardly a house so destitute as not to contain a newspaper. I have seen bands thus prepared that had been worn from four to six weeks, almost without a wrinkle; and so far as cording was concerned, it would have done to have used them a second time.

After the surgeon has seen that all of his apparatus is prepared, and has placed it where it can be conveniently obtained, then, but not till then, should he proceed to dress the limb. An assistant holding and steadying the knee, the surgeon applies two straps of adhesive plaster to each side of the leg from the tuberosities of the tibia to the maleoli, which are protected from undue pressure, by small pads placed over them. A pad is then placed under the leg just above the heel. It is held in place by bandages which extends from the toes and is carried evenly over the foot and leg to the knee. Experience has shown that in cases of fracture, the heel is exceedingly prone to ulceration, due to the partial arrest of circulation by the bandages, together with the long continued pressure from the weight of the leg. This pad is intended to be thick enough to prevent this pressure on the heel. The bandage also assists in retaining the plasters in their place, for, in warm weather especially, they are a little apt to slip when extension is made by them; also it prevents the swelling and stagnation of the blood, which necessarily would ensue if the thigh alone was bandaged. The perineal band is then carefully slid under the limb and brought to its proper place, the free ends extending above the hip. The long splint, which he has chosen, is then placed by the side of the limb, and a long pad, or junk, which reaches from the hip to the sole of the foot, is interposed between it and the leg. This pad should be made to fit the limb, being a little thicker opposite the knee and ankle. The ends of the band are carried through two holes in the upper end of the splint and tied together, and the free ends of the adhesive plaster are made fast to the extending apparatus in the foot-board, or if the splint is without any such apparatus, these ends are carried through two holes in the foot-board and firmly drawn down and tied together; in this case, whenever it is necessary to increase the extension, they must be untied and made shorter. The whole limb and splint must then be carefully raised by an assistant, while the surgeon places a splint underneath, one upon the inner side, and a third upon the upper side of the thigh. These splints should extend from the pelvis down to the knee, and should be so protected by pads, that neither the ends nor the sides of the splints shall come in contact with the skin. A bandage is then thrown around them, including the long splint, and as this bandage is carried down the limb, a large pad, three or more inches in thickness, is placed under the knee and thus held in place. The bandages used should be perfectly smooth, and applied to the limb with as few folds, or wrinkles, as is possible; for each fold is hard and apt to produce pain, if not ulceration. Sometimes the splints are held in place by four or more strips of bandage tied around them. This is convenient, if from any cause the surgeon wishes to open and examine the limb daily; or in case he expects considerable swelling of the thigh, he can readily loosen or tighten the bandages as the case may require. The bandage over the plaster, if well applied, will rarely require to be disturbed till that part of the treatment is dispensed with, which generally is about the fifth or sixth week. The object of the pad under the knee is to support that joint, and to prevent the leg from being perfectly extended. If a patient is placed upon his back with his limbs straightened out, without some support to this joint, in a few hours, intense pain will be produced, due to the unnatural straining of the flexor muscles of the leg. After the bandages are applied, and everything made comfortable, they should be basted together by a needle and thread. This will do much towards preventing their getting loose and displaced, and often prevent the necessity of a re-application of the bandages.

As a general rule, the patient should be seen every day for the first ten days after the accident. The surgeon should carefully examine the dressings, see that every part is right, that the splints have not become displaced, or that they do not cause pain by pressure, that the bandages are smooth and evenly applied, that they are neither too loose nor too tight, see that the extension and counter-extension is properly made, that it is not too great, that the skin is not becoming excoriated underneath the perineal band, that the heel is not painful from pressure, and finally, that the dressings are comfortable. It may seem but a little thing, but, nevertheless, it is of great importance that the surgeon should ascertain cause of pain, if any should be present, in a limb thus dressed, and if possible, remove it early; for not unfrequently, as often is the case with the heel, the pain may not be very severe, and lasting only from twelve to twenty-four hours, and then almost entirely disappearing; but when a few days after, the surgeon removes the dressings, he is surprised to find that sloughing has taken place, and that the fracture has become complicated with an ulcer, which becomes the source of great trouble, not only to himself, but also to his patient. If the dressings become loose or displaced, or if they cause pain, they should be taken off, and the limb re-dressed. With some patients this will have to be done quite often; a few will need to be re-dressed but a few times in the course of treatment, which generally is continued six weeks; then, if the union seems firm, the patient may be permitted to leave the bed, and go about with crutches.

Dr. Hamilton recommends, and as a general thing, uses the long, straight splint of Desault, in the footboard of which he has placed an endless screw, by means of which he makes extension. I myself have used the straight splint, with two holes made in the foot-board, through which the extending straps were carried, and then tied together. The best result that I have had in treating an oblique fracture of the femur, in an adult, was in the case of a man about 33 years of age, in which the amount of shortening was only one-quarter of an inch. This patient was treated by means of a long, straight splint, which I made out of a thin piece of clapboard, with a foot-board, rudely fashioned fıom a thicker piece, in a barn on the place. I made it, that I might dress the limb, and make the patient comfortable, till I should see him again; but on visiting him the next day, I found him quite comfortable, and the rude splint answering every purpose, and therefore did not change it.

